# Effects of Exergames on Physical Fitness in Middle-Aged and Older Adults in Taiwan

**DOI:** 10.3390/ijerph17072565

**Published:** 2020-04-08

**Authors:** Tzu-Cheng Yu, Che-Hsien Chiang, Pei-Tzu Wu, Wen-Lan Wu, I-Hua Chu

**Affiliations:** 1 Ph.D. Program in Biomedical Engineering, College of Medicine, Kaohsiung Medical University, Kaohsiung 807, Taiwan; u107805002@kmu.edu.tw; 2 Department of Sports Medicine, Kaohsiung Medical University, Kaohsiung 807, Taiwan; joseph3mo@gmail.com (C.-H.C.); wenlanwu@kmu.edu.tw (W.-L.W.); 3 Department of Rehabilitation, Oregon Health and Science University Hospital, Portland, OR 97239, USA; wuel@ohsu.edu; 4 Center for Long-Term Care Research, Kaohsiung Medical University, Kaohsiung 807, Taiwan; 5 Department of Medical Research, Kaohsiung Medical University Hospital, Kaohsiung 807, Taiwan

**Keywords:** exercise, muscle strength, flexibility, physical endurance, exergame

## Abstract

Using exergaming for exercise training was found to improve physical fitness. Yet, few studies have used the “Xbox Kinect” to examine its effects on physical fitness in healthy middle-aged and older adults. The purpose of this study was to investigate the effect of 10-weeks of Xbox Kinect training on physical fitness in healthy middle-aged and older adults. Forty participants (average 64.00 ± 4.44 years old, eight males and 32 females) were randomized to either intervention (n = 20) or control group (n = 20). The intervention group played Xbox Kinect three times per week, for an average of 50 min per session for 10 weeks. The control group was instructed to maintain their levels of physical activity. All the participants completed assessments of body composition, muscle strength, flexibility, balance and cardiopulmonary endurance at baseline and after 10-week intervention. After 10 weeks of training, the intervention group showed significant improvements in cardiopulmonary endurance and leg muscle strength. Moreover, there were significant differences between the intervention and control group in changes in aerobic fitness and leg muscle strength. The exergame program effectively improved cardiopulmonary endurance and leg muscle strength in healthy middle-aged and older adults. It could be an alternative to conventional exercise.

## 1. Introduction

Although health status declines with age [1,2], studies have found that improvement of physical fitness in the elderly can benefit overall physical function [3]. Regular exercise has been shown to be effective in improving physical fitness such as cardiopulmonary fitness, muscle endurance and balance, and reducing the incidence of cardiovascular disease, arthritis and other chronic diseases [4].

Among the several products of exergame (e.g., Nintendo Wii, Microsoft Xbox Kinect, Sony PlayStation), most studies were conducted using the Nintendo Wii. However, the Nintendo Wii requires a handheld device to play which may have limited the players’ physical performance. The Microsoft Xbox Kinect, on the other hand, does not require any handheld device to connect to the video games. Players can play video games through their body movements, such as jumping, squatting, running in place and side stepping [5]. Studies have suggested that players using the Microsoft Xbox Kinect can achieve light to moderate intensity of exercise [6,7]. Thus, exergames have been considered as a novel form of exercise [8,9]. Furthermore, compared with traditional exercise, exergames are more interesting and challenging, are not affected by the weather, and being able to perform the exercises at home increases safety and convenience [10].

While some research focused on the effects of exergames in children, teens or young adults [6], there are currently only three studies using the Xbox Kinect as an exercise intervention for healthy elderly people over 65 years old. However, no firm conclusion has been drawn on the effect of training by Xbox Kinect exergames on physical fitness in this population since these studies have adopted a variety of outcome measures, including balance ability, functional walking, quality of life, muscle strength, walking speed, and hand coordination [11,12,13]. In addition, few studies have investigated the effects of the Xbox Kinect on physical fitness in healthy middle-aged adults [6,10,14]. Therefore, the purpose of this study was to investigate the effect of 10-week Xbox Kinect exergame training on physical fitness in healthy middle-aged and older adults. We hypothesized that 10 weeks of exergame training would improve physical fitness in middle-aged and older adults.

## 2. Materials and Methods

### 2.1. Participants

**Figure 1:**
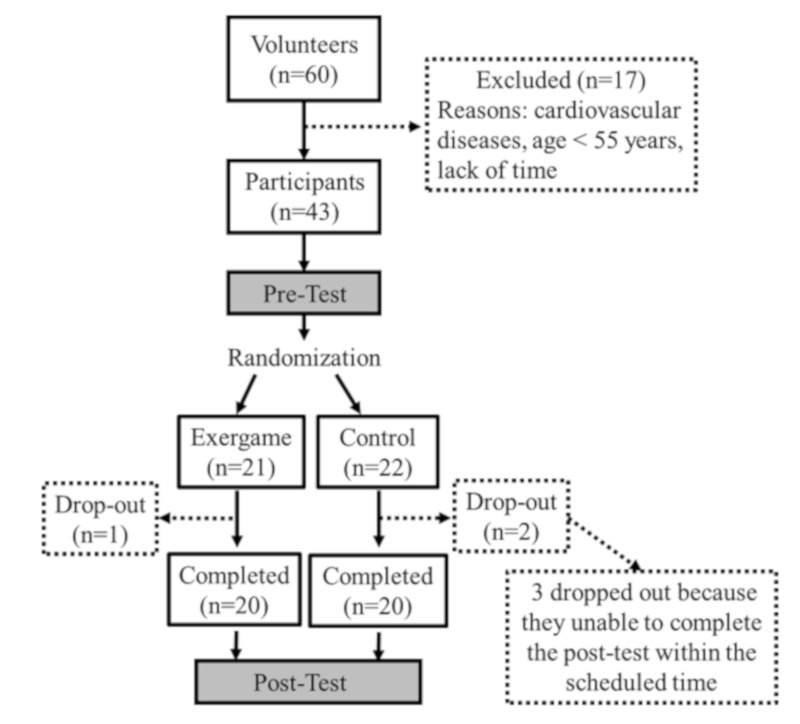
Flowchart of inclusion process.

Volunteers were recruited from the university and surrounding community via word-of-mouth, e-mails and posters. Among the 60 screened volunteers, 43 participants (55–76 years old) were recruited (Figure 1). The inclusion criteria were (1) age ≥ 55 years; (2) 18.5 ≤ BMI ≤ 29.9; (3) Mini-Mental State Examination (MMSE) ≥ 25. Volunteers were excluded if they (1) had musculoskeletal problems that may affect physical fitness tests and exercise training (e.g., joint pain or inflammation); (2) were unable to walk independently (e.g., using a walker); (3) had cardiovascular disease, diabetes, pulmonary disease or kidney disease; or (4) had any physical contraindications to perform exercise.

All the participants were informed about the purpose, procedure and precautions of the study before they were asked to sign and return the written Clinical Trial Informed Consent Form approved by the Kaohsiung Medical University Institutional Review Board (KMUHIRB-SV(II)-20150055) and were performed according to the Declaration of Helsinki.

### 2.2. Procedures

The eligible participants were randomly assigned to either (1) exergame group (EX; n = 21), or (2) control group (CON; n = 22) after completing the pre-test (Figure 1). 

The EX group underwent exergame training with an Xbox 360 Kinect^®^ (Xbox 360, Microsoft, Inc., Redmond, WA, USA) console for 10 weeks, 3 times a week and 50 min each time. The training session was conducted individually and supervised by research staff. Each exergame training session consisted of 10 min of warm-up, 30 min of exergame, and 10 min of cool-down. The participants in the CON group did not receive any intervention and were asked to maintain their lifestyle for 10 weeks. 

All participants performed a post-test after they completed the 10-week participation. The post-test was performed at least 72 hours after the last training session to avoid short-term effects of exercise training. 

Blood pressure (OMRON HEM-7200, OMRON HEALTHCARE Co., Ltd., Kyoto, Japan.) was measured before each training session, pre-test and post-test. If the participants’ systolic blood pressure ≥ 160 mmHg or diastolic blood pressure ≥ 110 mmHg at rest, exercise testing or training was postponed [15]. Participants’ heart rate was closely monitored (Mio Fuse, JoiiUp Technology Inc, Cochrane, AB, Canada) during each training session, pre- and post-test [16].

### 2.3. Exergames and Training Protocols

The Kinect Sports consisted of 6 exergames: boxing, beach volleyball, football, track and field, table tennis and bowling, while the Kinect Adventures required whole-body movements such as jumping over or dodging objects. The difficulty level of the games was adjusted according to the score of the last training session. 

During weeks 1, 3, 5, 7 and 9, participants’ weekly training protocol consisted of 2 exergame items of the Kinect Sports in each training session. Day 1: table tennis + football; Day 2: track & field + beach volleyball; Day 3: bowling + boxing. Each exergame item took 15 min to complete, resulting 30 min in total for each training session. The selection of exergame items was based on the intensity of exercise [7]. During weeks 2, 4, 6, 8 and 10, participants performed all 5 exergame items (3 min per item) of the Kinect Adventures and repeated once, resulting in a total of 30 min of training for each training day. 

### 2.4. Physical Activity Level

Physical activity level was measured before and after the intervention to monitor levels of physical activity in both groups using the self-administered Godin Leisure-time Exercise Questionnaire (GLEQ). Weekly strenuous (e.g., running), moderate (e.g., fast walking) and light (e.g., yoga) physical activities were recorded. A weekly leisure activity score was calculated by the following formula: 

Weekly leisure activity = (9 × Strenuous) + (5 × Moderate) + (3 × Light).

### 2.5. Body Composition

Body weight and body fat percentage at pre- and post-test were measured using a bioelectrical impedance analyzer (InBody 230, Biospace Co., Ltd., Chungcheongnam-do, Korea). Body height was also measured and the BMI was calculated (BMI = weight (Kg)/height squared (m^2^)).

### 2.6. Muscle Strength

Grip strength of the dominant hand was assessed with a hand-held dynamometer (TTM-YO, TTM, Tokyo, Japan) at pre- and post-test. Participants were asked to stand with feet shoulder-width apart. The tested arm was at the participants’ side with elbow flexed at 90 degrees. Participants were then encouraged to grasp and squeeze the hand-held dynamometer as hard as possible. Participants performed the grip strength test twice and the better score was recorded.

Leg strength was evaluated using the 30-second sit-to-stand test. Participants were seated on a chair with their back in an upright position. They were then instructed to look straight forward and repeatedly rise and sit down at their own preferred speed with their arms folded across their chest. Participants were encouraged to perform this motion as many times as possible within 30 seconds. 

### 2.7. Flexibility

The flexibility of the upper body and shoulder was measured using the back-scratch test [17]. They were instructed to place the dominant hand behind the head and back over the shoulder with the palm touching the back and the fingers pointing downward, then place the other hand behind the back with the palm facing outward, fingers pointing upward and reaching up as far as possible. The distance between the tips of the middle fingers was measured. If the fingertips did not meet, the score was assessed as a negative difference of the fingertip gap distance. 

The flexibility of the back and leg was assessed by the chair sit-and-reach test as described [18]. Participants were seated on the edge of a chair, and instructed to keep their left foot flat on the floor while their right leg was extended forward with the knee straight, heel on the floor, and ankle bent at 90 degrees. With the extended leg and spine as straight as possible and hands on top of each other (tips of the middle fingers even), participants were encouraged to reach forward slowly toward the toes by bending at the hip. The distance between the tips of the middle fingers and the toes was measured. If the fingertips did not reach the toes, a negative score was recorded. 

Each flexibility test was performed twice and the better result was recorded.

### 2.8. Balance Ability

The static balance ability was assessed using a force plate system (Type 9286AA, Kister Instrument Corp., Winterhur, Switzerland). Participants were asked to stand barefoot on their preferred leg, with the other leg raised so that the raised foot was near but not touching the ankle of their stance leg. Participants were also asked to focus on a spot on a wall at eye level in front of them while maintaining the one-leg stance for 30 seconds. During the test, the center of pressure (COP) movement was recorded and the total length of the path of COP was calculated. 

The dynamic balance ability was assessed by the 8-foot up-and-go test [19]. Participants were asked to get out of a chair, walk 8 feet toward and around a cone, and return to the chair in the shortest time possible. 

Each participant performed each balance test twice and the better result was used for analysis.

### 2.9. Cardiopulmonary Endurance

The six-minute walk test (6MWT) was completed according to the American Thoracic Society guidelines [20]. The 6MWT was performed along a straight, flat enclosed 20-meter corridor. The starting line and the turnaround point were both marked with a cone. Then, each participant was asked to walk back and forth along the corridor at his or her own pace for 6 min. The distance covered over the 6 min was recorded.

### 2.10. Statistical Analyses

Descriptive statistics were calculated for all variables at pre-test (baseline) and post-test. The independent t test and the χ2 test were performed to examine any difference between groups at pre-test. Repeated measures analyses of variance (ANOVA), using a Group × Time mixed model, were performed to examine differences in all variables after 10 weeks of intervention. Partial Eta squared (η2) was used as an indicator of effect size. Significance was determined as a 2-tailed α value of less than 0.05. All statistical analyses were performed using the Statistical Package for the Social Sciences Version 19 (SPSS, IBM Corporation, Chicago, IL, USA).

## 3. Results

Among the 43 participants, three participants dropped out and 40 participants (64 ± 4.4 years of age; eight males and 32 females) completed the study (Figure 1). No significant difference in participant’s characteristics was observed at pre-test between the EX and CON groups (Table 1).

The average heart rate of the EX group during exercise training was 120.13 ± 14.80 beats per minute (bpm), with maximum heart rate at 180 bpm and minimum heart rate at 100 bpm. The heart rate reserve during the training session was 50.67 ± 15.93%, indicating that on average the participants were exercising at moderate intensity. The attendance rate of the exergame training sessions was 79.67 ± 10.65%. 

### 3.1. Weekly Physical Activity Level

After 10 weeks, the physical activity duration, frequency and intensity were significantly increased in the EX group (all *p* < 0.01; Table 2) while no significant difference was observed in the CON group. There was a significant interaction between group and time for the weekly physical activity level (all *p* < 0.01).

### 3.2. Body Composition

There were no interactive or main effects of group and time on BMI or body fat in the EX or CON group (Table 2). The results indicate that no significant changes in either BMI or body fat occurred in either group.

### 3.3. Muscle Strength

There was no significant difference in the grip strength within or between EX and CON groups (Table 2). Leg strength was significantly increased after 10 weeks of intervention in the EX group (*p* = 0.001) while no significant difference was observed in the CON group (*p* = 0.714). There was a significant interaction between group and time for leg strength (*p* = 0.012; Table 2)

### 3.4. Flexibility

There were no interactive or main effects of group and time on either upper body (back-scratch test) or back and leg (sit-and-reach test) flexibility in the EX or CON group (Table 2). No significant change in flexibility was observed in both groups after 10 weeks.

### 3.5. Balance Ability

After 10 weeks, there were no interactive or main effects of group and time on either static or dynamic balance in the EX or CON group (Table 2). Both groups showed no significant changes in balance ability after 10 weeks.

### 3.6. Cardiopulmonary Endurance

After 10 weeks, the distance of the 6MWT significantly increased in the EX group (*p* = 0.001), but there was no significant difference in the CON group (*p* = 0.350). There was a significant interaction between group and time for the 6MWT (*p* = 0.025; Table 2).

## 4. Discussion

The purpose of present study was to investigate the effect of Xbox Kinect intervention on physical fitness in middle-aged and older adults. The main finding of the present study was that the Xbox Kinect exergame training effectively improved the physical activity level, cardiopulmonary endurance and leg strength of healthy middle-aged and older adults, although no significant effect was observed on BMI, body fat, grip strength, flexibility, and balance ability. 

According to the classification of intensity score [21], the exercise group in the present study was improved from moderate (score = 14~23) to highly active (score ≥24). Both duration and frequency of leisure time exercise also increased significantly in the exercise group. In addition, the exercise intensity during exergaming was 50% of the heart rate reserves (HRR), which is within the American College of Sports Medicine’s recommended moderate intensity range for aerobic exercise training (i.e., 40%–60% HRR) [22]. These findings indicate that the Xbox Kinect was able to effectively increase the level of physical activity and provide adequate exercise training intensity.

Previously published studies have shown that exergame training improved the distance of the 6MWT by 14%~17% using the Wii sports console [23,24], compared to that of 5.5% in the current study. The discrepancy could be due to the fact that the participants in the present study were younger (64 vs. 74 years old) and generally in better health. In fact, the distance of the 6MWT in the present study was greater by 100 meters at baseline, compared to the published studies [23,24]. According to van Heuvelen et al.’s study [25], walking endurance contributed independently and significantly to the prediction of disability in the elderly. Therefore, the results of the present study suggest that older adults may improve their walking endurance and prevent or reduce disability by playing exergames.

The findings of the 30-second sit-to-stand, a surrogate test for leg strength, demonstrate an improvement by 15%, which is similar to previously published studies. Maillot et al. discovered that after 12 weeks of Wii Sports training, the number of 30-second sit-to-stand test increased by 21% in elderly people [23]. Orsega-Smith et al. found that by using Wii Fit sports training, 30-second sit-to-stand test performance was improved by 16% after as little as 4 weeks of training in overweight older adults [26]. A large number of studies have confirmed that exercise promotes the improvement of the skeletal muscle system [27], while the improvement of muscle strength in the elderly is mainly due to the increased ability of motor neurons and their activated muscle fibers to recruit motor units [28].. Interestingly, studies have suggested that lower extremity strength is strongly associated with cardiopulmonary fitness [24,29,30], which explains the significant improvement of both 6MWT and 30-second sit-to-stand in the present study.

The present study found consistent results where Ordnung et al. reported that no change was observed in grip strength after 6 weeks of Kinect training in healthy elderly people [11]. However, Jungok et al. presented opposite findings whereby grip strength increased by 6.5% in elderly women after 8 weeks of Kinect training [12]. The discrepancy may be due to the younger and male participants included in the present study, who may require higher intensity of exercise to elicit improvement in grip strength. In addition, the design of the exergame used in the present study seemed to involve more leg than arm movements, which could also potentially lead to no significant change in the grip strength. 

While previous studies reported improvement in static balance abilities, the present study did not find similar results. This can be explained by the differences in equipment used for measurements. Previous studies used the Wii Balanceboard© [11] or the Berg Balance Scale [31], while balance ability was evaluated using a force plate system in the present study. For the dynamic balance ability, the time for the 8-foot up-and-go in the present study was improved by approximately 5% although not statistically significant (*p* = 0.06). This finding was comparable to some studies which reported 8~13% improvements for the 8-foot up-and-go in elderly adults [13,23]. The finding of no significant change in flexibility is in agreement with previous studies [12,15,32], and may have resulted from the design of the games used, which do not focus on stretching muscles.

Research in recent years has suggested that effective balance training should combine integrated sports with cognition involvement [33]. The implementation of exergames requires the recruitment of multiple levels of cognitive ability as well as a great level of physical control involvement. Therefore, exergames may be one of the best choices for training balance ability. 

In the body composition part, the results show that after Xbox Kinect exergame training, the participants’ BMI and body fat ratio did not improve. While previous research on exergames did not observe changes in BMI or body fat percentage, leg muscle mass was reported to have increased in older adults [32]. Research has demonstrated that dietary control and regular physical activities were able to improve body composition in elderly people [34]. Furthermore, high-intensity training has reported to effectively lower subcutaneous and visceral fat [16]. Therefore, exergame training may be able to improve body composition if coupled with dietary control and/or higher intensity of game setting. Interestingly, some studies reported that exergame training alone lowered BMI or body fat percentage in children [35,36] and women [37].

The limitations of present study are: (1) the sample size of the study was relatively small and the gender distribution was not ideal. In addition, the participants in the present study have relatively better physical fitness than the average middle-aged and older adults. Therefore, the findings of this study may not be generalized to all middle-aged and older people; (2) exergame training requires a certain level of cognition to successfully perform physical movements. Therefore, participants’ understanding of the game rules may affect the results of exergame training; (3) the averaged HRR (ranged between 26% and 82%) in the present study has reached moderate intensity; however, participants’ familiarity with the game, effort and movement accuracy could affect the exercise intensity. 

The findings of this study can potentially be applied to future studies involving home-based Xbox Kinect exergaming, which would be relatively practical and closer to real-life in order to facilitate regular physical activities, compared to laboratory-based interventions. Moreover, it may be possible to add other physical fitness testing items (e.g., more balance assessments, and other upper limb muscle strength assessments, etc.) in future studies to have a more comprehensive perspective of the effect of Xbox Kinect training on physical fitness in healthy middle-aged and older adults.

## 5. Conclusions

The Xbox Kinect exergames were able to provide moderate intensity of exercise and increase physical activity level in middle-aged and older adults. Furthermore, exergaming also improved cardiorespiratory endurance and leg muscle strength after 10 weeks of training. Exergames are fun and can be played at home, which may mitigate the effects of time and environmental barriers to exercise. The results of the present study suggest that Xbox Kinect exergames could potentially be an alternative to conventional exercise, and ultimately improve physical fitness in healthy middle-aged and older adults.

## Figures and Tables

**Table 1:** Group comparison at pre-test.

Variables	All (n=40)	EX (n=20)	CON (n=20)	*p*-Value
Age (years)	64.00 ± 4.44	64.25 ± 4.75	63.75 ± 4.23	0.727
Male (N; %)	8 (20%)	5 (25%)	3 (15%)	0.429
SBP (mmHg)	134.25 ± 19.57	139.40 ± 21.69	129.10 ± 16.11	0.096
DBP (mmHg)	76.50 ± 11.35	79.55 ± 10.80	73.45 ± 11.33	0.089
Heart rate (bpm)	73.25 ± 9.36	72.80 ± 9.81	73.70 ± 9.13	0.766
MMSE	27.77 ± 1.46	28.10 ± 1.33	27.45 ± 1.54	0.162
Weekly leisure activity level				
Duration (min/week)	292.13 ± 266.45	296.75 ± 294.53	287.50 ± 242.77	0.914
Frequency (times/week)	4.13 ± 2.62	4.20 ± 2.48	4.05 ± 2.82	0.859
Intensity score	20.25 ± 18.77	19.45 ± 14.13	21.05 ± 22.85	0.791
Body composition				
BMI (kg/m^2^)	23.27 ± 2.73	23.26 ± 2.74	23.28 ± 2.79	0.978
Body fat (%)	30.55 ± 7.76	28.55 ± 9.06	32.55 ± 5.75	0.105
Muscle strength				
Grip strength (kg)	26.58 ± 7.71	27.30 ± 8.30	25.85 ± 7.20	0.559
Sit-to-stand (times/30sec)	17.68 ± 4.92	18.55 ± 5.37	16.80 ± 4.40	0.267
Flexibility				
Back-scratch (cm)	2.38 ± 7.72	3.40 ± 7.30	1.35 ± 8.18	0.408
Sit-and-reach (cm)	9.80 ± 13.95	7.75 ± 17.60	11.85 ± 8.99	0.359
Balance ability				
Static balance (cm)*	25.98 ± 4.33	25.69 ± 3.94	26.33 ± 4.89	0.689
Dynamic balance (sec)	5.23 ± 0.66	5.10 ± 0.55	5.35 ± 0.75	0.236
6MWT (m)	544.38 ± 66.24	554.35 ± 59.76	534.40 ± 72.30	0.348

Data are mean ± SD. SBP - systolic blood pressure; DBP - diastolic blood pressure; MMSE - Mini-Mental State Examination; BMI - body mass index. * means a total of 31 subjects (EX = 17, CON = 14) completed the static balance test.

**Table 2:** Outcome variables.

Variables	EX (n = 20)		CON (n = 20)		Time × Group p-Value	η^2^
	Pre-Test	Post-Test	Pre-Test	Post-Test		
SBP (mmHg)	139.40 ± 21.69	135.25 ± 19.64	129.10 ± 16.11	127.75 ± 14.31	0.474	0.014
DBP (mmHg)	79.55 ± 10.80	75.75 ± 10.23*	73.45 ± 11.33	72.80 ± 10.96	0.204	0.042
Heart rate (bpm)	72.80 ± 9.81	74.05 ± 9.32	73.70 ± 9.13	73.55 ± 10.92	0.605	0.007
Weekly leisure activity level						
Duration (min/week)	296.75 ± 294.53	436.25 ± 280.50*	287.50 ± 242.77	273.00 ± 198.39	0.007	0.177
Frequency (times/week)	4.20 ± 2.48	5.95 ± 1.32*	4.05 ± 2.82	3.95 ± 2.70	0.004	0.195
Intensity score	19.45 ± 14.13	31.85 ± 13.85*	21.05 ± 22.85	18.20 ± 14.88	0.004	0.201
Body composition						
BMI (kg/m2)	23.26 ± 2.74	23.33 ± 2.67	23.27 ± 2.79	23.29 ± 2.78	0.562	0.009
Body fat (%)	28.55 ± 9.06	29.25 ± 8.08	32.55 ± 5.75	32.41 ± 5.68	0.188	0.045
Muscle strength						
Grip strength (kg)	27.30 ± 8.30	27.00 ± 10.57	25.85 ± 7.20	24.60 ± 6.82	0.385	0.020
Sit-to-stand (times/30sec)	18.55 ± 5.37	21.40 ± 5.26*	16.80 ± 4.40	17.05 ± 5.62	0.012	0.154
Flexibility						
Back-scratch (cm)	3.40 ± 7.30	3.60 ± 8.64	1.35 ± 8.18	1.75 ± 7.88	0.828	0.001
Sit-and-reach (cm)	7.75 ± 17.60	7.35 ± 15.38	11.85 ± 8.99	11.20 ± 9.05	0.829	0.001
Balance ability						
Static balance (cm) †	25.69 ± 3.94	25.88 ± 5.10	26.33 ± 4.89	26.77 ± 3.72	0.815	0.002
Dynamic balance (sec)	5.10 ± 0.55	4.85 ± 0.67	5.35 ± 0.75	5.30 ± 1.13	0.346	0.023
6MWT (m)	554.35 ± 59.76	584.65 ± 53.27*	534.40 ± 72.30	539.20 ± 84.03	0.025	0.126

Data are mean ± SD. SBP - systolic blood pressure; DBP - diastolic blood pressure; MMSE - Mini-Mental State Examination; BMI - body mass index. * indicate significant difference between pre-test and post-test (P < 0.05). †means total of 31 subjects (EX = 17, CON = 14) completed the static balance test.
